# Structural Transformation to Attain Responsible BIOSciences (STARBIOS2): Protocol for a Horizon 2020 Funded European Multicenter Project to Promote Responsible Research and Innovation

**DOI:** 10.2196/11745

**Published:** 2019-03-07

**Authors:** Vittorio Colizzi, Daniele Mezzana, Pavel V Ovseiko, Giovanni Caiati, Claudia Colonnello, Andrea Declich, Alastair M Buchan, Laurel Edmunds, Elena Buzan, Luiz Zerbini, Dimitar Djilianov, Evanthia Kalpazidou Schmidt, Krzysztof P Bielawski, Doris Elster, Maria Salvato, Luiz C Jr Alcantara, Antonella Minutolo, Marina Potestà, Elena Bachiddu, Maria J Milano, Lorna R Henderson, Vasiliki Kiparoglou, Phoebe Friesen, Mark Sheehan, Daniela Moyankova, Krasimir Rusanov, Martha Wium, Izabela Raszczyk, Igor Konieczny, Jerzy P Gwizdala, Karol Śledzik, Tanja Barendziak, Julia Birkholz, Nicklas Müller, Jürgen Warrelmann, Ute Meyer, Juliane Filser, Fernanda Khouri Barreto, Carla Montesano

**Affiliations:** 1 Department of Biology University of Rome Tor Vergata Rome Italy; 2 Radcliffe Department of Medicine University of Oxford Oxford United Kingdom; 3 Laboratorio di Scienze della Cittadinanza Rome Italy; 4 Department of Biodiversity, University of Primorska Koper Slovenia; 5 International Centre for Genetic Engineering and Biotechnology Cape Town South Africa; 6 Agrobioinstitute, Agricultural Academy Sofia Bulgaria; 7 Danish Centre for Studies in Research and Research Policy, University of Aarhus Aarhus Denmark; 8 Intercollegiate Faculty of Biotechnology, University of Gdańsk and Medical University of Gdańsk Gdańsk Poland; 9 Faculty of Biology & Chemistry, University of Bremen Bremen Germany; 10 University System of Maryland Baltimore, MD United States; 11 Fundacao Oswaldo Cruz Rio de Janeiro Brazil; 12 Department of History, Humanities and Society, University of Rome Tor Vergata Rome Italy; 13 Oxford University Hospitals National Health Service Foundation Trust Oxford United Kingdom; 14 Nuffield Department of Population Health, University of Oxford Oxford United Kingdom; 15 Faculty of Management, University of Gdansk Gdańsk Poland

**Keywords:** action plans, ethics, gender, guidelines, Horizon 2020, institutional change, model for RRI in biosciences, open access, patient involvement, public engagement, responsible research and innovation, structural change, science education, science with and for Society

## Abstract

**Background:**

Promoting Responsible Research and Innovation (RRI) is a major strategy of the “Science with and for Society” work program of the European Union’s Horizon 2020 Framework Programme for Research and Innovation. RRI aims to achieve a better alignment of research and innovation with the values, needs, and expectations of society. The RRI strategy includes the “keys” of public engagement, open access, gender, ethics, and science education. The Structural Transformation to Attain Responsible BIOSciences (STARBIOS2) project promotes RRI in 6 European research institutions and universities from Bulgaria, Germany, Italy, Slovenia, Poland, and the United Kingdom, in partnership with a further 6 institutions from Brazil, Denmark, Italy, South Africa, Sweden, and the United States.

**Objective:**

The project aims to attain RRI structural change in 6 European institutions by implementing action plans (APs) and developing APs for 3 non-European institutions active in the field of biosciences; use the implementation of APs as a learning process with a view to developing a set of guidelines on the implementation of RRI; and develop a sustainable model for RRI in biosciences.

**Methods:**

The project comprises interrelated research and implementation designed to achieve the aforementioned specific objectives. The project is organized into 6 core work packages and 5 supporting work packages. The core work packages deal with the implementation of institutional APs in 6 European institutions based on the structural change activation model. The supporting work packages include technical assistance, learning process on RRI-oriented structural change, monitoring and assessment, communication and dissemination, and project management.

**Results:**

The project is funded by Horizon 2020 and will run for 4 years (May 2016-April 2020). As of June 2018, the initial phase has been completed. The participating institutions have developed and approved APs and commenced their implementation. An observation tool has been launched by the Technical Assistance Team to collect information from the implementation of APs; the Evaluation & Assessment team has started monitoring the advancement of the project. As part of the communication and dissemination strategy, a project website, a Facebook page, and a Twitter account have been launched and are updated periodically. The International Scientific Advisory Committee has been formed to advise on the reporting and dissemination of the project’s results.

**Conclusions:**

In the short term, we anticipate that the project will have a considerable impact on the organizational processes and structures, improving the RRI uptake in the participating institutions. In the medium term, we expect to make RRI-oriented organizational change scalable across Europe by developing guidelines on RRI implementation and an RRI model in biosciences. In the long term, we expect that the project would help increase the ability of research institutions to make discoveries and innovations in better alignment with societal needs and values.

**International Registered Report Identifier (IRRID):**

DERR1-10.2196/11745

## Introduction

The misalignment of research and innovation with society could negatively affect the European Research Area in a number of ways [1]. First, European research and innovation could become unable to address the key problems that European society is facing and therefore, to contribute to “achieving objectives of sustainable development (consisting of economic, social, as well as environmental aspects)” [2]. Second, European research and innovation could become unable to exploit its potential in terms of innovation and commercial impacts and consequently, less competitive in the global market. Third, European research and innovation could become socially isolated, ethically contested, and not supported by citizens, public authorities, and economic players, with negative consequences for the availability of public research funds and private investments [3,4].

Fundamentally, what is at stake is the capacity of European research to be relevant and effective in societal and economic terms. In response, promoting Responsible Research and Innovation (RRI) is a major strategy of the “Science with and for Society” work program of the European Union’s Horizon 2020 Framework Programme for Research and Innovation [5].

The term ”Responsible Research and Innovation”, while increasingly popular over the past decade [6-10], is conceptually underdeveloped and inconsistently applied [11]. According to the European Commission (EC), the RRI approach should be a key part of the research and innovation process and should be established as a collective, inclusive, and system-wide approach. In practice, RRI is implemented by the EC as a package that includes multiple actors and public engagement in research and innovation, enabling easier access to scientific results, the take-up of gender and ethics in the content and process of research and innovation, and both formal and informal science education [12].

In the literature, RRI is viewed as an “umbrella term” comprising a series of theoretical approaches and methods, and cutting across different sectors. As such, a wide range of stakeholders are involved in the RRI governance, which can be characterized as a patchwork of different and sometimes shared responsibilities. Most of the analyzed studies aim to contribute to the development of RRI in a specific discipline or area of research, drawing attention to the sedimented nature of the concept [13].

The aim of the Structural Transformation to Attain Responsible BIOSciences (STARBIOS2) project funded by the European Union’s Horizon 2020 research and innovation program (grant agreement No 709517) is to contribute to the advancement of the RRI strategy underpinning Horizon 2020. The specific objectives of the project are to attain RRI structural change in 6 European institutions through the implementation of action plans (APs) and to develop APs for 3 non-European institutions active in the field of biosciences; to use the implementation of APs as a learning process to develop a set of guidelines on the implementation of RRI; and to develop a sustainable model for RRI in biosciences

The STARBIOS2 project has been designed and is currently being carried out to promote RRI in 6 European institutions from Bulgaria, Germany, Italy, Slovenia, Poland, and the United Kingdom in partnership with a further 6 institutions from Brazil, Denmark, Italy, South Africa, Sweden, and the United States. The project is coordinated by the University of Rome Tor Vergata.

## Methods

### Theoretical Approach

The conceptual framework underpinning STARBIOS2 includes 4 major thematic elements, which are the interpretation of RRI; the relevance of RRI to the bioscience research sector; the interpretation of structural change; and the activation of structural change processes in research institutions and universities.

#### The Interpretation of Responsible Research and Innovation

**Figure 1:**
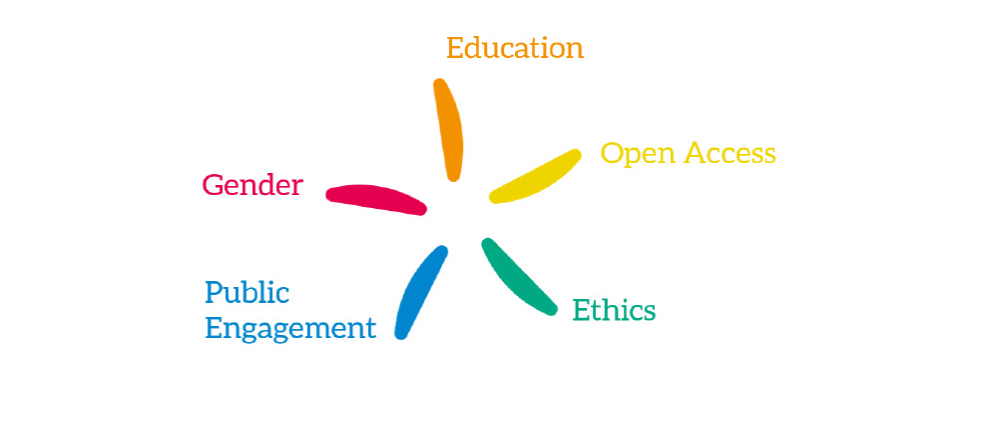
Five keys of Responsible Research and Innovation as depicted in the Structural Transformation to Attain Responsible BIOSciences logo.

In the context of this project, RRI is interpreted as an overarching policy strategy to radically increase the intensity and quality of the interactions between the European Research Area and European society. RRI aims to achieve a better alignment of research and innovation with the values, needs, and expectations of society [14]. At the level of research institutions, RRI activates structural processes able to profoundly modify their culture, values, rules, and procedures in 5 key areas (Figure 1):

- *Public engagement*: promoting the engagement of all societal actors—including researchers, citizens, policy makers, persons involved in business and industry, school children, and teachers—with the research and innovation process
- *Gender*: improving the excellence of scientific research, advancing gender equality within research institutions, as well as within the design and content of research and innovation
- *Education*: enhancing current educational strategies to provide future researchers and other societal actors with new capacities for taking responsibility in the research and innovation process and attracting children and youth to science, technology, engineering, mathematics, and medicine
- *Open Access*: making research and innovation transparent, free of charge, and easily accessible online, without restriction
- *Ethics*: ensuring that research and innovation respects fundamental rights and ethical standards, and shifting the view of research ethics away from a process of constraint, to one of supporting high-quality research results.

Although interest in some of the RRI issues, especially gender equality, ethics, and public engagement, is not new at European, national, and institutional levels, the novelty of the STARBIOS2 project is in bringing together the 5 keys of RRI in a unique strategy supported by a robust governance framework. Another important feature that differentiates STARBIOS2 from other RRI projects is the multicenter application of RRI in the field of biosciences. Another important differentiating feature of STARBIOS2 is the attempt to produce sustainable structural changes in the participating institutions.

#### Relevance of Responsible Research and Innovation to the Bioscience Research Sector

While RRI aims to increase the intensity and quality of interactions between research and society in general, such interactions vary according to the research sector. Different research sectors have developed specific ways and methods for interacting with society with respect to the specific sectorial scientific, economic, and social challenges being faced [15], the societal players involved and their demands for engagement, communication styles, relationships with industry and, broadly, intensity of and tools for interaction with society. Furthermore, the uptake of RRI markedly impacts the contents and methods of research and innovation, which vary from sector to sector. Moreover, different disciplinary communities tend to interpret RRI according to their specific contexts, and attempts have already been made to develop RRI models tailored to specific research sectors. STARBIOS2 focuses on one specific research sector, biosciences, which can be understood in a broad sense to include biomedicine, biology, system biology, biochemistry, nature conservation, and biotechnology sciences [16]. The STARBIOS2 approach to RRI is relevant to address the diverse and complex challenges that arise in biosciences, including challenges related to public awareness of epidemics and vaccines, social responsibility for global nutrition, open access to big data, etc. Furthermore, the STARBIOS2 approach to RRI encourages collaboration across disciplinary boundaries and regions, as well as between scientific and nonscientific communities. This type of collaboration is increasingly required to deal with novel bioscience concepts such as the exposome, that is, all the biological, environmental, and social exposures relevant to an individual’s health.

#### The Interpretation of Structural Change

In the context of the EC research policy environment, structural change refers to profound modifications of universities and research organizations [17] to pursue definite policy objectives. The concept of structural change is akin to the concept of institutional transformation [18] as both are based on the assumption that, in some cases or under given circumstances, the pursuit of new objectives requires pervasive, intensive, and far-reaching changes of the fundamental organizational processes, setup, and values [19]. Based on the experience of gender equality policies in science and technology [20-26], structural change is often characterized by 4 main features:

- *Irreversibility*: induced transformations are so rooted in the institution that they cannot be easily reversed (eg, by a simple leadership turnover or budget cuts)
- *Comprehensiveness*: a modification of the organizational life, affecting cultural and cognitive attitudes of staff and leaders, daily behaviors and practices, communication patterns [27], as well as procedures, rules, standards, and organizational structure
- *Inclusiveness*: structural change, as a collective effort, has to involve all players and stakeholders within the involved institutional or organizational unit, from leadership to students; therefore, both top-down and bottom-up processes are to be activated and coordinated
- *Contextualization*: even though problems and situations can be highly recursive and widespread, their mix is unique; this creates a need to contextualize structural change, for example, devising strategies and tools that are specifically tailored to the institution or unit

#### The Activation of Structural Change Processes in Research Institutions

**Figure 2:**
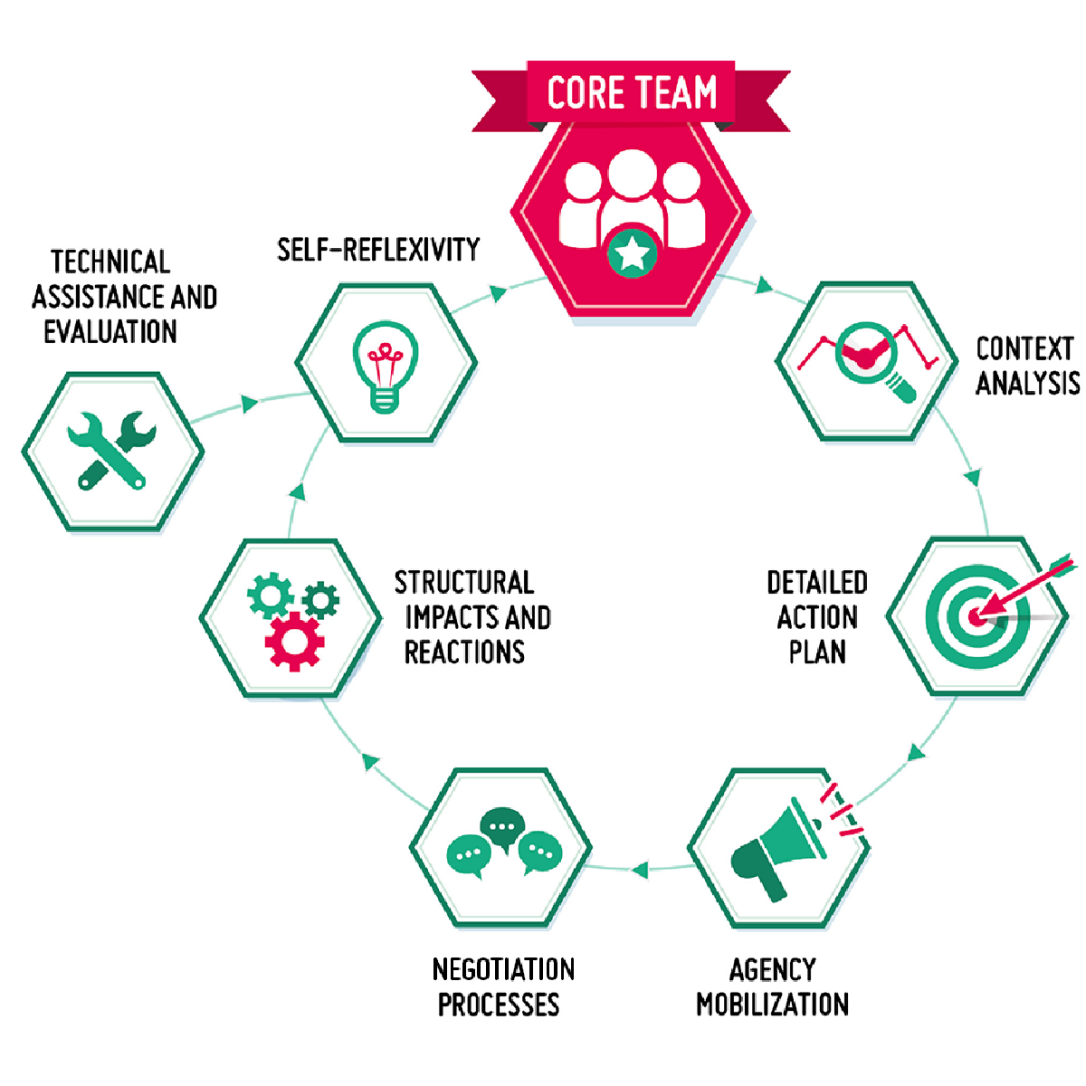
A structural change activation model adapted from the European Commission-funded project STAGES.

Structural change will be activated through the design and implementation of institutional APs based on the iterative model adapted from the EC-funded project STAGES [28] (Figure 2).

The building blocks of the structural change activation model are as follows:

- *Core team*: In each research institution involved in the project, a core team is established to be in charge of the AP implementation. This core team is a source of new agency oriented to activate structural change processes [29-31] toward RRI within the department and the institution as a whole.
- *Context analysis*: One of the first actions of the core team is to analyze the context for the development and implementation of the AP. The context analysis considers multiple aspects, including previous experience within the department and the research institution in the key areas of RRI; the actors to be involved, their attitudes and orientations toward RRI, as well as their willingness to cooperate in the implementation of the AP; and the existing norms, organizational arrangements, and procedures applied in the management of each of the 5 RRI areas.
- *Detailed AP*: Based on the general AP included in the project’s description of work and in the light of the context analysis, the core team develops a detailed AP.
- *Agency mobilization*: The core team is engaged with other influential individuals, groups, or networks already oriented toward or willing to promote RRI to enlarging the team and mobilizing them on RRI.
- *Negotiation processes*: Any action activates a negotiation process. Usually, negotiations related to consensus building or leadership on RRI involve multiple dimensions, including the interpretive dimension, which involves the interpretation of the situation within the organization, the symbolic dimension, which includes the visibility and recognition of RRI and its components, the institutional dimension, which pertains to the actual modification of the institutional structure, and the operational dimension, which translates decisions, goodwill, and declarations into action.
- *Structural impacts and reactions*: Negotiation processes are expected to have structural impacts on the institutions involved. Initial structural impacts will serve as the foundation upon which the rest will be built. At the same time, structurally negative reactions may also occur, changing the context or requiring modifications to the AP.
- *Self-reflexivity*: The core team is required to be self-reflective, that is, well aware of objectives, obstacles, timelines, opportunities, facilitating factors, and risks.
- *Technical assistance and monitoring and assessment*: The design and implementation of institutional APs is supported through technical assistance and monitoring and assessment activity provided by 2 partners.

This model describes the nature of the AP implementation phase. In biosciences and other contexts characterized by high levels of uncertainty, innovation, and social complexity, project implementation processes rarely assume a linear trajectory. Rather, they tend to be nonlinear, characterized by stops and starts, sudden progress and setbacks, unplanned solutions, and deviations from the original plan. As a corollary, the implementation phase will require proactivity, flexibility, and the capacity to react rapidly to unexpected situations.

### Project Partners

The STARBIOS2 project has been designed and is currently being carried out in 6 European institutions as follows: The Department of Biology, University of Rome Tor Vergata, Rome, Italy; the National Institute for Health Research Oxford Biomedical Research Centre, University of Oxford and Oxford University Hospitals NHS Foundation Trust, Oxford, United Kingdom; the Department of Biodiversity, University of Primorska, Koper, Slovenia; The Institute of Science Education, University of Bremen, Bremen, Germany; the Agrobioinstitute, Sofia, Bulgaria; and the Intercollegiate Faculty of Biotechnology University of Gdańsk and Medical University of Gdańsk, Gdańsk, Poland.

In the context of the global significance of RRI, the STARBIOS2 project is carried out in partnership with 6 additional institutions worldwide.

The three partners play roles in developing their own APs and the learning and sharing of RRI implementation experience and best practice beyond Europe are The International Centre for Genetic Engineering and Biotechnology, Cape Town, South Africa, The Oswaldo Cruz Foundation, Rio de Janeiro, Brazil, and The University of Maryland, Baltimore, MD, United States.

Another 3 partners have responsibilities for providing the project consortium with the following contributions:

- The Laboratory of Citizenship Sciences, Rome, Italy (responsible for technical assistance)
- The Danish Centre for Studies in Research and Research Policy, Aarhus University, Aarhus, Denmark (responsible for monitoring and assessment)
- The Centre for Research Ethics & Bioethics, Uppsala University, Sweden (responsible for communication and dissemination; in 2018, this partner replaced Sparks & Co [France], which oversaw the communication activities in the first phase of the project).

### Project Structure

**Figure 3:**
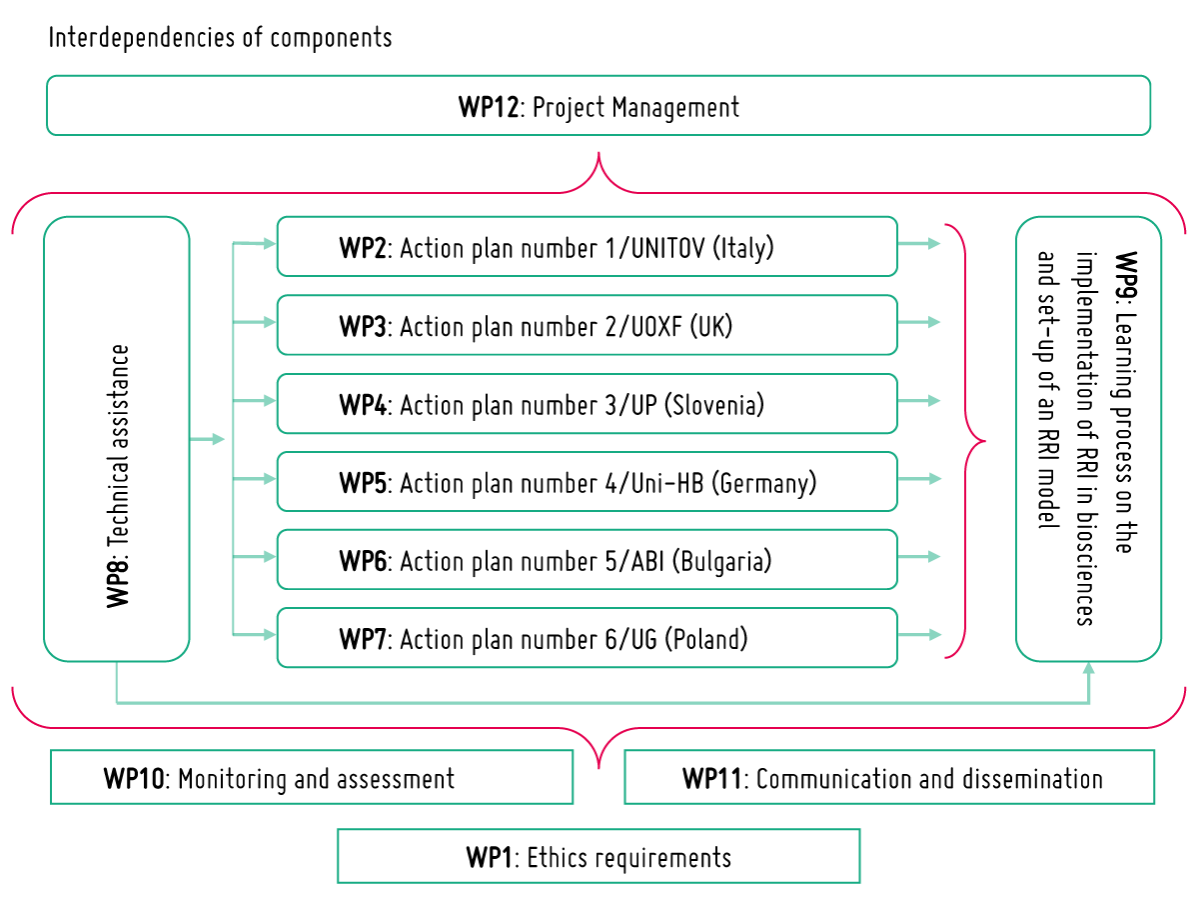
The project organization and management. RRI: Responsible Research and Innovation; UNITOV: University of Rome Tor Vergata; UOXF: University of Oxford; UP: University of Primorska; Uni-HB: University of Bremen; ABI: Agrobioinstitute; UG: University of Gdańsk; UK: United Kingdom; WP: work package.

The project is organized into 6 core work packages dealing with the implementation of institutional APs based on the structural change activation model and others supporting work packages (Figure 3).

The work packages related to the 6 APs have in common the methodology illustrated above, and the fact that they deal with all 5 keys of RRI; these work packages, however, differ in the fact that they are designed to meet different local needs and operate in different political, cultural, and scientific contexts.

While each partner is responsible for the delivery of their specific APs, services, and learning activities related to the work packages, the overall project management is based on the principle of coresponsibility. Coresponsibility enables equal participation of consortium members in key decisions over the course of the project and underpins the composition of the Steering Committee, which includes representatives of all partners. The Project Coordinator presides over the Steering Committee and coordinates the project at the executive and scientific levels. A lead organization has been identified for every work package. In close cooperation with work package leaders, the Project Coordinator and their staff monitor the execution of the project, including risk analysis and adjustments to the original plan of activities. In addition to the internal controls that the Steering Committee has for the scientific and technical quality of the activities, the project also has an International Scientific Advisory Committee, an independent body providing external oversight of the scientific quality of the main outputs of the project.

### Technical Assistance and Evaluation

This specific project work package aims to provide continuous support to the partners involved in the implementation of the project APs through technical assistance activities in all phases of the project. These activities are expected to help drive the APs successfully from the detailed design phase to completion and ensure that the implementation of each AP can benefit from other RRI experiences in bioscience worldwide. Technical assistance is carried out through regular consultations via Skype, on-site visits, email correspondence, meeting on specific thematic issues with one or more AP teams, and mutual learning sessions during the Steering Committee meetings.

Different types of technical assistance include horizon scanning and structured presentation of materials, scientific texts, manuals, video, and events relevant for the different keys areas of RRI; self-reflection activities; horizontal exchanges among teams; facilitation of contact with external experts on specific issues foreseen within the different APs; promotion of contact between the experts inside the consortium; administrative and technical advice on financial and reporting issues; suggestions for tools to be used or adopted for carrying out specific activities; and support in designing and setting up specific actions.

### Monitoring and Assessment

Working in cooperation with the Technical Assistance Team, the Monitoring and Assessment Work Package aims to examine and assess the process and progress toward the objectives of the project, provide input on the quality of the project activities, and assess the achievement of planned objectives and impacts, in acknowledgment and adaptation to the project nature [32]. Specific monitoring and assessment activities include the following:

- Transversal: cooperation with all other partners, facilitation of knowledge exchange, and technical assistance;
- Communication: identification of needs and potential beneficial activities, inducing critical self-reflection; and
- Balancing an internal or external role: a critical friend, overseeing the flow of the APs, mapping progress, and enabling timely intervention.

### Development of the Guidelines and Model

RRI APs at STARBIOS2 institutions serve as “laboratories” of structural change; they will allow us to carry out guided observations and develop learning processes related to the implementation of RRI. Therefore, the project will allow us to learn lessons on how to foster a process of structural change oriented toward RRI in the bioscience sector. The authors will synthesize these lessons in a set of guidelines on the implementation of RRI within bioscience research organizations and develop a model for the diffusion of such activities in the bioscience sector. These activities will be carried out following a participatory approach.

The guidelines will contain recommendations for initiatives to promote structural change within research organizations aimed at promoting RRI. The model, on the other hand, will be focused on the general characteristics of RRI for a research organization within the bioscience sector.

The following activities are planned: information-gathering (through technical assistance and monitoring, literature review, and exchanges with other projects on RRI); analysis of existing RRI models; second-tier analysis of the STARBIOS2 APs; drafting of the guidelines provisional version; validation and final version of the guidelines; development and validation of a model for implementing RRI in the biosciences structure; drafting of a strategic document for a sustainable RRI model for biosciences; training program on RRI in biosciences with the involvement of STARBIOS2 international partners; and development of RRI APs by our partner institutions from South Africa, Brazil, and the United States.

### Interconnections Between Activities

All project activities are interconnected with each other into 3 major components, where the outcomes of each preceding component serve as preconditions to the outcomes of each subsequent component:

- The implementation of the 6 APs (together with the related support, technical assistance, and monitoring activities) is directly intended to produce structural changes in the institutions involved.
- The development of the learning process is dependent upon the attainment of structural changes through the implementation of the APs, as well as comparisons between the experiences of implementing various APs.
- The development of the model on RRI for biosciences and the guidelines is, in turn, intrinsically dependent on the outcomes of the learning process.

In the long term, it is expected that the model and the guidelines developed and disseminated as part of this project will help to increase the ability of research institutions to make discoveries and innovations in better alignment with societal needs and values.

### Communication and Dissemination

The results of this project will be communicated and disseminated through the professional networks of the partner institutions, conferences, workshops, peer-reviewed journals, trade publications, and mass media. To increase transparency and broaden outreach, research news and multimedia will also be published online on the project website, social networks, and social media.

### Ethics

As part of the current European Union’s Horizon 2020 research and innovation program (grant agreement No. 709517), all partners completed the required ethics self-assessment and confirmed that all activities raising ethical issues would comply with applicable international, European, and national law; formally, all publicly funded bioscience institutions should require that. Ethics approval will be sought from the relevant Research Ethics Committees and host institutions at the appropriate time prior to commencing individual research components of projects involving human participants or personal data. Ethical breaches should be punishable by the loss of public research funding and often by civil law suits.

## Results

The project runs for 4 years from May 2016 to April 2020. As of June 2018, the initial phase has been completed. The 6 APs have been approved by the partners and are ongoing. All management, support, and advisory structures have been activated, and are working on a regular basis. A specific approach for mutual learning has been developed and discussed with all the AP Teams. The work on the development of a set of guidelines on the implementation of RRI and a model for RRI in biosciences has commenced and is currently at an early stage. An International Scientific Advisory Committee has been formed to advise on the reporting and dissemination of the project’s results. As part of the communication and dissemination strategy, a project website [33] a Facebook page, and a Twitter account [34] have been launched and are updated periodically.

In addition, an ongoing observation tool has been launched; this observation tool, managed mainly by the Technical Assistance Team, will enable the collection of information from AP activities, which will be useful for the elaboration of guidelines and model. Aspects to be taken into consideration, in the development of the guidelines and model include the following: the mobilization of the actors and their agency within the process of implementing RRI-oriented initiatives and projects; the barriers encountered during the implementation of RRI-oriented initiatives and projects; the negotiation in which the actors are engaged in during the promotion of RRI; the structural impacts and reactions produced by the RRI promotion process; how self-reflection is carried out by the actors involved; and how context impacts research organizations and the promotion of RRI.

The substantive phase of the project is currently in full progress. The first substantive results include the following:

- The launch under the aegis of the United Nations Educational, Scientific and Cultural Organization Interdisciplinary Chair in Biotechnology and Bioethics of a university course on “Wellness, food and sustainable development”; the launch of an Observatory on gender to raise awareness on how gender models affect research activity and to modify organizational models (University of Rome Tor Vergata)
- The adoption of an Open Access Policy by the University Senate; the promotion of courses and theses about career development for women researchers; and the inclusion of RRI in the XXIV Biotechnology Summer School as a “pilot” experience toward the establishment of a teaching course in the educational curricula (University of Gdańsk)
- The creation of a Code of Conduct for Biosciences and its implementation into the syllabus of 3 courses; the adoption of gender among the quality indicators for the self-assessment of the activities implemented by different university departments (University of Primorska, Koper)
- The establishment of a Plant Biotechnology Information Centre aimed at promoting societal engagement and science communication around emerging research, ethical, and societal issues and the establishment of contacts with public institutions and nongovernmental organizations (Agrobioinstitute, Sofia)
- The elaboration of educational methods and tools to promote the awareness of RRI in different target groups (students, researchers, school classes, teachers, and citizens) and the development of an RRI mission statement at faculty level (University of Bremen)
- The evaluation and promotion of the use of sex and gender as key variables in biomedical research; the facilitation of knowledge exchange about open access among researchers and practitioners via presentations, digital strategies, and policy consultations (University of Oxford and Oxford University Hospitals NHS Foundation Trust).

## Discussion

The authors anticipate that the project will have a significant impact on the organizational processes and structures of the institutions involved through the uptake of RRI. The authors expect to make RRI-oriented organizational change scalable across Europe through the development of a set of guidelines on the implementation of RRI and a model for RRI in biosciences. In the long term, the authors expect that the project will help increase the ability of research institutions to make discoveries and innovations in better alignment with societal needs and values. Potential risks within the delivery and impact of the project include the complexity of the project, which might affect the planned timeline for the delivery of the project, unexpected responses to changes in organizational culture, procedures, and structures, and the management of the involvement of a large number of stakeholders during various phases of the project. These potential risks are being addressed by the adoption of a context-specific approach to the implementation of project APs in different contexts, adequate stakeholder engagement, constant communication among the partners, periodic monitoring and assessment of the project delivery against its objectives, and continuous technical assistance throughout all phases of the project provided by one of the partners. Cooperation with international partners allows the learning and sharing of RRI implementation experience and best practice beyond Europe.

## Abbreviations

AP: action plan
EC: European Commission
RRI: Responsible Research and Innovation
STARBIOS2: Structural Transformation to Attain Responsible BIOSciences

## Appendix

Multimedia Appendix 1 Evaluation Summary Report Coordination and support actions.
