# Supplementary material for: Structural Transformation to Attain Responsible BIOSciences (STARBIOS2): Protocol for a Horizon 2020 Funded European Multicenter Project to Promote Responsible Research and Innovation
Source: JMIR Res Protoc. 2019 Mar 7;8(3):e11745. doi: 10.2196/11745 (PMC6427101; doi:10.2196/11745)
Supplement: Multimedia Appendix 1 [file resprot_v8i3e11745_app1.pdf]

Ref. Ares(2016)330569 - 21/01/2016

Proposal Evaluation Form

EUROPEAN COMMISSION<br>Horizon 2020 - Research and Innovation Framework Programme	**Evaluation Summary Report - Coordination and support actions**

**Call:**	H2020-ISSI-2015-1
**Funding scheme:**	Coordination & support action
**Proposal number:**	709517
**Proposal acronym:**	STARBIOS 2
**Duration (months):**	48
**Proposal title:**	Structural Transformation to Attain Responsible BIOSciences
**Activity:**	ISSI-5-2015

N.	Proposer name	Country	Total Cost	%	Grant Requested	%
1	UNIVERSITA DEGLI STUDI DI ROMA TORVERGATA	IT	807,522	23.08%	807,522	23.08%
2	LABORATORIO DI SCIENZE DELLA CITTADINANZA - LSC	IT	324,750	9.28%	324,750	9.28%
3	THE CHANCELLOR, MASTERS AND SCHOLARS OF THE UNIVERSITY OF OXFORD	UK	413,338	11.81%	413,338	11.81%
4	UNIVERZA NA PRIMORSKEM UNIVERSITA DEL LITORALE	SI	275,938	7.89%	275,938	7.89%
5	AGROBIOINSTITUTE	BG	318,760	9.11%	318,760	9.11%
6	INTERNATIONAL CENTRE FOR GENETIC ENGINEERING AND BIOTECHNOLOGY	IT	126,250	3.61%	126,250	3.61%
7	AARHUS UNIVERSITET	DK	155,000	4.43%	155,000	4.43%
8	UNIWERSYTET GDANSKI	PL	312,370	8.93%	312,370	8.93%
9	UNIVERSITAET BREMEN	DE	347,000	9.92%	347,000	9.92%
10	SPARKS & CO	FR	225,100	6.43%	225,100	6.43%
11	University System of Maryland	US	132,000	3.77%	132,000	3.77%
12	FUNDACAO OSWALDO CRUZ	BR	61,250	1.75%	61,250	1.75%
	Total:		3,499,278		3,499,278	

Abstract:
STAR BIOS 2 (Structural Transformation to Attain Responsible BIOSciences),coordinated by the University of Tor Vergata (IT), has been designed to respond to the Topic ISSI 5 (Workprogramme Science With And For Society). The general aim of project is that of contributing to the advancement of the Responsible Research and Innovation (RRI) strategy, which underpins Horizon 2020, by promoting 6 Action Plans (APs) oriented to attain a RRI structural change in research institutions from Europe and developing 3 further APs in non-european entities, all active in the field of biosciences. This strategy is geared to cope more in general with one of the main risk, for European research, i.e., its inadequate connection with society, by promoting its increasing alignment, in terms of both process and outcomes, with the needs and values of European society. This entails, in the RRI perspective, an increasing involvement of stakeholders at any level of the research and innovation process. The project has three main focuses: 1) Develop RRI-oriented structural change processes in the already mentioned institutions involved in biosciences research. This aim will be pursued through designing, implementing and evaluating RRI Action Plans. In order to secure the results emerging from the APs, a sustainability strategy will be developed and implemented during the project lifespan. APs will be supported by a central technical assistance and the project will be monitored and assessed. 2) Develop a learning process concerning: a) resistances and barriers to RRI (which are they, how they manifest themselves, which impact they have, etc.); b) key factors favouring or supporting RRI; c) strategic options and RRI-oriented tools. 3) Develop a sustainable model for RRI in biosciences.

Evaluation Summary Report

Evaluation Result

Total score: 13.00 (Threshold: 10)

Form information

SCORING

Scores must be in the range 0-5.

Interpretation of the score:

*0– The **proposal fails to address the criterion** or cannot be assessed due to missing or incomplete information.*

*1– **Poor.** The criterion is inadequately addressed, or there are serious inherent weaknesses.*

*2– **Fair.** The proposal broadly addresses the criterion, but there are significant weaknesses.*

*3– **Good.** The proposal addresses the criterion well, but a number of shortcomings are present.*

*4– **Very good.** The proposal addresses the criterion very well, but a small number of shortcomings are present.*

*5– **Excellent.** The proposal successfully addresses all relevant aspects of the criterion.Any shortcomings are minor.*

Criterion 1 - Excellence

Score: **4.00** (Threshold: 3/5.00 , Weight: -)

Note: The following aspects will be taken into account, to the extent that the proposed work corresponds to the topic description

in the work programme. If a proposal is partly out of scope, this must be reflected in the scoring, and explained in the comments.
Clarity and pertinence of the objectives

Objectives are described in detail, and are clearly outlined, measurable and verifiable. They are relevant to the call description and to the H2020 cross-cutting issues. The RRI keys, which are central in the call, are very convincingly integrated in the proposal.

Credibility of the proposed approach

The proposed approach is credible and feasible because it develops in local action plans and projects, embedded in each institutional context, building on existing experience, movements and opportunities.

Soundness of the concept

The integration of local institutional practices and various cultural approaches is very coherent and it will result in valuable guidelines based on institutional experiences and observations.

The trans-disciplinary considerations are sufficiently addressed.

However, the analysis of the concept is insufficiently developed; it rather describes how experiences in the participating institutes could be analysed as a result of the project.

Quality of the proposed coordination and/or support measures

The proposed coordination and support measures are well described and very good, certainly benefiting from partners and the coordinator experience, who have implemented numerous projects within the scope of FP5, FP6 and FP7 programs. The project will lead to many lessons learned and a demonstration of best practices, which can be shared with other institutes.

Criterion 2 - Impact

Score: **4.00** (Threshold: 3/5.00 , Weight: -)

Note: The following aspects will be taken into account, to the extent to which the outputs of the project should contribute at the European and/or International level:
The expected impacts listed in the work programme under the relevant topic

The proposal's expected impacts correspond to the relevant topics listed in the work programme.

The project will result in a well-described and evaluated set of best practices and alternative approaches, making it a valuable tool-kit for institutions throughout Europe and the world.

However, the expected impact to making institutional change scalable is overestimated because the project is not starting from, or working towards a clear model, rather several locally based models.

Effectiveness of the proposed measures to exploit and disseminate the project results (including management of IPR), to communicate the project, and to manage research data where relevant

The proposal contains a detailed plan of dissemination and communication of its expected outcomes in the biosciences field. On the very concrete level of institutions, the proposal will result in positive change and create awareness for change, providing examples for other researchers and institutes in Europe and the world that could be followed.

The communication plan is very promising and will reach and influence its audiences. However, considering the two main objects to be disseminated, namely Guidelines for RRI implementation and the RRI model for bioscience, some aspects of the effectiveness are insufficiently addressed: exhibitions and participatory events are not noted; the number of people to be reached by collecting email addresses among partners and snowballing is too low; and estimated number of people attending the final events doesn't offer significant impact.

Criterion 3 - Quality and efficiency of the implementation

Score: **5.00** (Threshold: 3/5.00 , Weight: -)

Note: The following aspects will be taken into account:
Coherence and effectiveness of the work plan, including appropriateness of the allocation of tasks and resources

The work plan is well defined, coherent and effective in its work packages and milestones. The list of deliverables is impressive. The description of the organisational structure and budget of the project is adequate.

Within the consortium there is a good distribution of the work, some partners are directly involved in the implementation of the action plan, others in contributing to its exploitation at the international level, and still others, to transversal actions. The allocation of tasks between the partners is well suited. The distribution of resources is appropriate.

The risk of the work packages drifting apart, because of their locally based specificities, is overcome by the interaction between them and the work packages for technical assistance, evaluation, communication, learning processes and project management, increasing the efficiency and effectiveness of the project.

Complementarity of the participants within the consortium (when relevant)

The 12 participants in the consortium are complementary to each other. Within the consortium there is a good distribution of the work, some partners are directly involved in the implementation of the action plan, others in contributing to its exploitation at the international level, and still others, to transversal actions. The Europeans partners are geographically well organized and the international partners are diverse.

However, the consortium lacks sufficient integration of organisations representing civil society, special interest groups and policy makers, who could add valuable extra input and reflection to the proposed approach.

The individual participants are well qualified in their fields. There is a good balance among the research, educational and social sciences.

Appropriateness of the management structures and procedures, including risk and innovation management

The management structure and procedures are appropriate in terms of work distribution, participatory decision-making and verification. There

are also adequate provisions for risks arising from conflicts, innovativeness, complexity, openness and the participatory approach. The project will benefit from the extensive project management experience of the partners.

Scope of the proposal

Status: **Yes**

Comments (in case the proposal is out of scope)

Not provided

Operational Capacity

Status: **Operational Capacity: Yes**

If No, please list the concerned partner(s), the reasons for the rejection, and the requested amount.

Not provided

Exceptional funding of third country participants/international organisations

A third country participant/international organisation not listed in General Annex A to the Main Work Programme may exceptionally receive funding if their participation is essential for carrying out the project (for instance due to outstanding expertise, access to unique know-how, access to research infrastructure, access to particular geographical environments, possibility to involve key partners in emerging markets, access to data, etc.). (For more information, see the Online Manual)

Based on the information provided in the proposal, we consider that the following participant(s)/international organisation(s) that requested funding should exceptionally be funded:
(Please list the Name and acronym of the applicant, Reasons for exceptional funding and the Requested grant amount.)

University System of Maryland (US), Fundação Oswaldo Cruz (Brazil) and ICGEB (International organization).
They will provide international experience from outside the EU.

Based on the information provided in the proposal, we consider that the following participant(s)/international organisation(s) that requested funding should NOT be funded:
(Please list the Name and acronym of the applicant, Reasons for exceptional funding and the Requested grant amount.)

Not provided

Use of human embryonic stem cells (hESC)

Does this proposal involve the use of hESC?

No

If yes, please state whether the use of hESC is, or is not, in your opinion, necessary to achieve the scientific objectives of the proposal and the reasons why. Alternatively, please also state if it cannot be assessed whether the use of hESC is necessary or not because of a lack of information.

Not provided

Overall comments

It is noted in this resubmission that the composition of the consortium has changed and the technical annexes have been modified.
